# Traumatic posterior sternoclavicular joint dislocation in a child: a case report

**DOI:** 10.11604/pamj.2014.19.386.5684

**Published:** 2014-12-17

**Authors:** Gabriel Ngom, Azhar Salim Mohamed, Mohamed Ould El Housseine, Oumar Ndour

**Affiliations:** 1 Unit of Pediatric Surgery, University Hospital Aristide Le Dantec, Dakar, Senegal

**Keywords:** Sternoclavicular joint, child, dislocation, CT scan, closed reduction

## Abstract

Sternoclavicular joint dislocation is a rare event. It occurs most often in a violent trauma. The authors report the case of a10 years old child, received at emergencies for right shoulder blunt trauma after been punched by another child. He presented with right shoulder pain, right upper limb functional impairment and right sternoclavicular joint depression. Standard chest radiographs were normal. Chest CT scan showed posterior dislocation and allowed us to determine its variety. Twelve hours after the trauma, a closed reduction has been done under general anesthesia. A control CT scan showed a restoration of normal joint anatomy. After 18 months, the shoulder was painless and mobile in all directions. It is an isolated recent posterior sternoclavicular joint dislocation in a child. With this observation the authors emphasizeon the unusual mechanism of such a dislocation occurrence, the primary role of CT scan in the diagnosis and early closed reduction.

## Introduction

Sternoclavicular joint dislocation (SCJD) is a rare injury. It represents, in fact, only 1% of all dislocation in humans and 3% of shoulder dislocations [1]. It occurs most often during a sports accident, traffic road accident or a fall from a height [2]. The SCJD may be anterior or posterior, the latter representing only 5% of cases [3]. The latter form can be too severe and may be fatal. We report a case of a SCJD in its posterior from in a child. The objective of this work was to discuss the mechanism, diagnosis and treatment compared with literature data.

## Patient and observation

**Figure 1:**
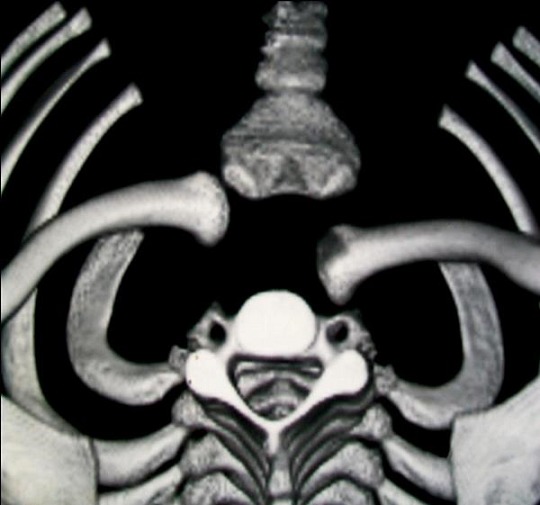
Chest CT scan showing right posterior sternoclavicular joint dislocation

**Figure 2:**
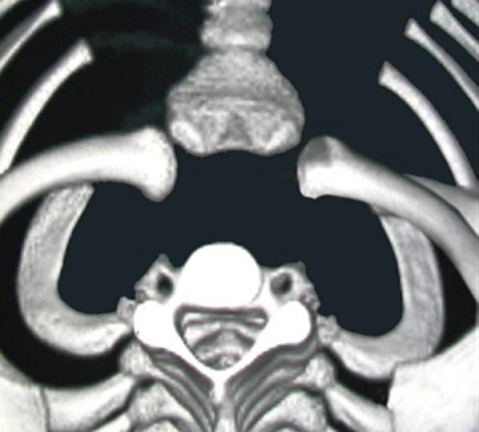
Chest CT scan showing reduction

A 10 years old male child right-handed, was received on May 15, 2011 in HALD Pediatric Surgery Department for the right shoulder blunt trauma, 7 hours before admission. He had been punched on the right anterior shoulder by another child, older than him. He then felt pain and developed anterior chest deformity and right upper limb relative functional impairment. The child received before admission a massage from a traditional healer without any success. On physical examination, he presented a good general status, conscience, well colored conjunctiva and a temperature of 37°C. There were also a depression and a provoked pain on the right sternoclavicular joint. The mobilization of the right shoulder was painful. There were neither downstream neurovascular disorders, no respiratory or gastrointestinal symptoms. AP chest radiography was normal. Chest CT scan showed a right SCJD in its posterior form (Figure 1). A closed reduction under general anesthesia had been done 12 hours after the trauma. It was obtained by traction in adduction applied on the arm in the axis of the upper limb with a direct manipulation of the clavicle medial end. The reduction was confirmed by an audible snap. The distal pulses were palpable and a figure-eight bandage was done. A control chest CT scan showed a reduction of the SCJD (Figure 2). The radial pulse, motor and sensitive function of the right upper limb were evaluated and the child was discharged after two days of hospitalization. The figure-eight bandage was removed after six weeks of immobilization. After a flow-up of 18 months, the right shoulder was painless and mobile in all directions.

## Discussion

In posterior dislocation direct mechanism is dominant [4]. It is often the direct force impact applied to the medial part of the clavicle with the arm flexed and adducted. The force that produces the SCJD is often violent, due to a sports injury, the road traffic accident or a fall from a height. In our case, the mechanism is direct. But the force that led to the subsequent SCJD is linked to a punch given by a child, therefore a low-velocity trauma, witch is an exceptional situation. The shoulder pain is the key symptom in the SCJD and is often severe and accompanied by limited shoulder movements [5]. Sometimes, the patient present with an abnormal voice, breathing or swallowing difficulties which are associated with mediastinal involvement [5]. The clinical examination found the shoulder depression in SCJD [5]. This depression may be absent in cases of edema in the region [5]. A palpatory pain is noted. Rare but life-threatening complications should be systematically sought: dyspnea, dysphagia, upper limb paralysis, bleeding general signs [6–8]. These complications are related to the presence of certain structures in the posterior mediastinum: the arteriosustruncus and innominate vein, the vagus nerve, the phrenic nerve, the internal jugular vein, trachea and esophagus. Plain radiographs, including the Serendipity view, are difficult to perform and interpret [2, 5]. Even if they show asymmetry of both clavicles, indirect sign of dislocation, they can not formally establish the diagnosis; specify the anterior or the posterior dislocation variety. Currently, the SCJD is best explored by CT scan, by making fine and joined cuts [9]. It can not only appreciate the dislocation and its variety but also study the surrounding soft tissues. In posterior form, the injection is imperative. It allows analyzing the precise relationship between the medial end of the dislocated clavicle and the mediastinum [7].

In our patient, it is an isolated posterior dislocation of the right sternoclavicular joint. The clinical signs of the SCJD were present. However, we could not exclude a physeal fracture of the medial end of the clavicle. Children have more physeal fractures than dislocations. Plain radiographs were not contributive in our patient. It is the chest CT scans that revealed the lesion and highlight the treatment. In recent and isolated posterior dislocations, closed reduction under general anesthesia is required within 48 hours to have the maximum chance of success. The reduction technique consists of placing the patient in supine position with a sandbag between the two shoulder blades. Traction is made on the adducted arm, while a pressure on the shoulder to the back is applied. If external maneuvers fail, towel clip or a small sharp bone holding forceps placed percutaneous can help reduction [3]. The presence of a thoracic surgeon is recommended for possible vascular complications [10]. Woman and leagus have indeed reported the existence of dry wound in the right pulmonary artery blocked by the clavicle displacement, which bled during reduction [8]. In recent posterior dislocations with closed reduction failure or the existence of chest complications, surgical treatment is needed while avoiding the pins that can migrate and cause dramatic complications, due to the proximity of the mediastinum. In any case, restraint is provided by a figure-eight bandage which must be maintained for 4-6 weeks. In our patient, closed reduction under general anesthesia was justified. Indeed, it was a recent dislocation with an early diagnosis without any associated complication. However, the collaboration with a thoracic surgeon, as recommended in the literature, was lacking. In the recent posterior SCJD, clinical signs and chest scan allow an early diagnosis and to look for possible complications related to the posterior mediastinum. If treatment is orthopedic in isolated SCJD, surgery is indicated for closed reduction failure or complications.

## Conclusion

With this observation the authorsemphasizeon the unusual mechanism of such a dislocation occurrence, the primary role of CT scan in the diagnosis and early closed reduction.
